# Bis{2-[(2,4,6-trimethyl­phen­yl)imino­methyl]pyrrol-1-ido}palladium(II)

**DOI:** 10.1107/S1600536813000573

**Published:** 2013-01-12

**Authors:** Wolfgang Imhof

**Affiliations:** a University Koblenz-Landau, Institute for Integrated Natural Sciences, Universitätsstrasse 1, 56070 Koblenz, Germany

## Abstract

The title compound, [Pd(C_14_H_15_N_2_)_2_], is a square-planar palladium complex composed of two deprotonated pyrrole-2-carbaldimine ligands coordinating a central Pd^II^ atom. In the crystal, three crystallographically independent complex mol­ecules are observed, one of which is located in a general position, whereas the Pd^II^ atoms of the other mol­ecules are situated on crystallographic inversion centers. The aromatic substituents at the imine N atoms in the three mol­ecules show dihedral angles of 87.6 (7)/83.64 (7), 74.3 (7) and 88.3 (7)° with respect to the corresponding PdN_4_ plane.

## Related literature

For structural analyses of the related ligand *N*-((1*H*-pyrrol-2-yl)methyl­ene)aniline, see: Gomes *et al.* (2010 ▶); Crestani *et al.* (2011 ▶) and of of the free ligand *N*-((1*H*-pyrrol-2-yl)methyl­ene)-2,4,6-trimethyl­aniline, see: Imhof (2013 ▶). For the structure of the corresponding nickel complex, see: Anderson *et al.* (2006 ▶), the closely related 2,6-dimethyl complex, see: Pérez-Puente *et al.* (2008 ▶), the closely related 2,6-diisopropyl complex, see: Liang *et al.* (2004 ▶) and a related nickel complex with only one pyrrole-carbaldimine ligand, see: Bellabarba *et al.* (2003 ▶).
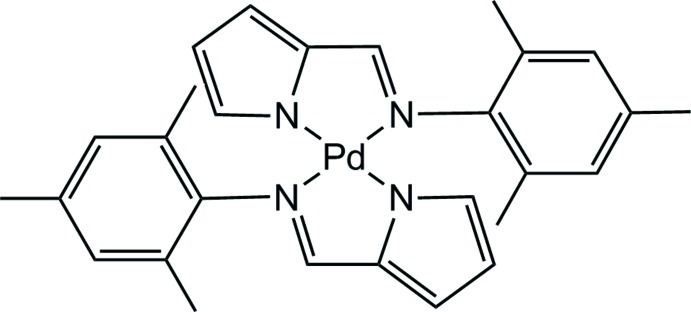


## Experimental

### 

#### Crystal data

[Pd(C_14_H_15_N_2_)_2_]
*M* *_r_* = 528.96
Triclinic, 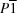

*a* = 13.4342 (2) Å
*b* = 13.7395 (3) Å
*c* = 13.9932 (3) Å
α = 89.174 (11)°
β = 77.075 (12)°
γ = 77.159 (12)°
*V* = 2452.76 (19) Å^3^
*Z* = 4
Mo *K*α radiation
μ = 0.78 mm^−1^
*T* = 183 K
0.3 × 0.3 × 0.2 mm

#### Data collection

Nonius KappaCCD diffractometer
18633 measured reflections
11178 independent reflections
9176 reflections with *I* > 2σ(*I*)
*R*_int_ = 0.025

#### Refinement

*R*[*F* ^2^ > 2σ(*F* ^2^)] = 0.032
*wR*(*F* ^2^) = 0.082
*S* = 1.04
11178 reflections
610 parameters
H-atom parameters constrained
Δρ_max_ = 1.17 e Å^−3^
Δρ_min_ = −0.80 e Å^−3^

### 

Data collection: *COLLECT* (Nonius, 1998 ▶); cell refinement: *DENZO* (Otwinowski & Minor, 1997 ▶); data reduction: *DENZO*; program(s) used to solve structure: *SHELXS97* (Sheldrick, 2008 ▶); program(s) used to refine structure: *SHELXL97* (Sheldrick, 2008 ▶); molecular graphics: *ORTEP-3 for Windows* (Farrugia, 2012 ▶); software used to prepare material for publication: *publCIF* (Westrip, 2010 ▶).

## Supplementary Material

Crystal structure: contains datablock(s) I, global. DOI: 10.1107/S1600536813000573/nc2300sup1.cif

Structure factors: contains datablock(s) I. DOI: 10.1107/S1600536813000573/nc2300Isup2.hkl

Additional supplementary materials:  crystallographic information; 3D view; checkCIF report

## Figures and Tables

**Fig. 1.**
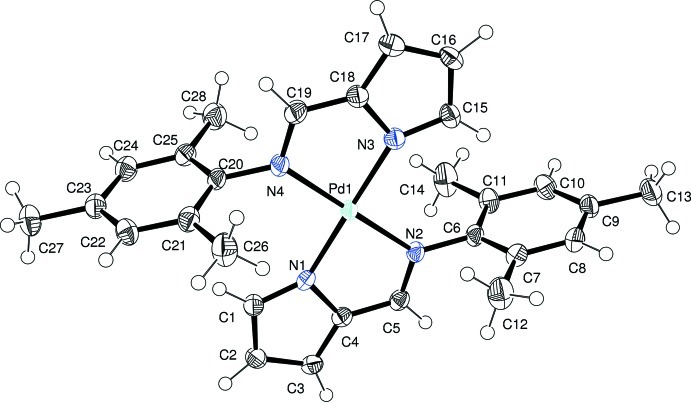
Molecular structure of the molecule in general positions in the unit cell with thermal ellipsoids at the 50% probability level.

**Fig. 2.**
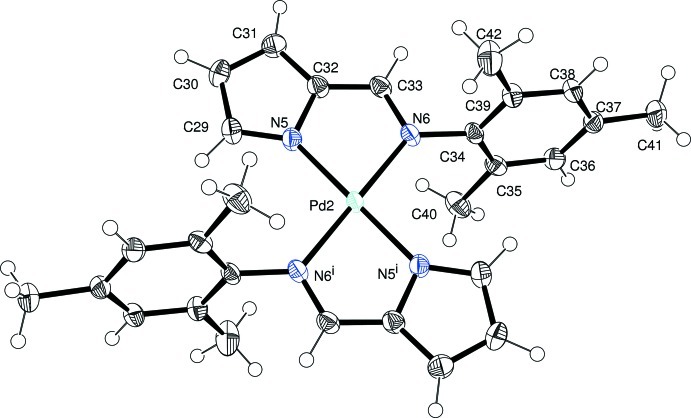
Molecular structure of the molecule with Pd2 being situated at a inversion center with thermal ellipsoids at the 50% probability level (i = -x, -1 - y, -z).

**Fig. 3.**
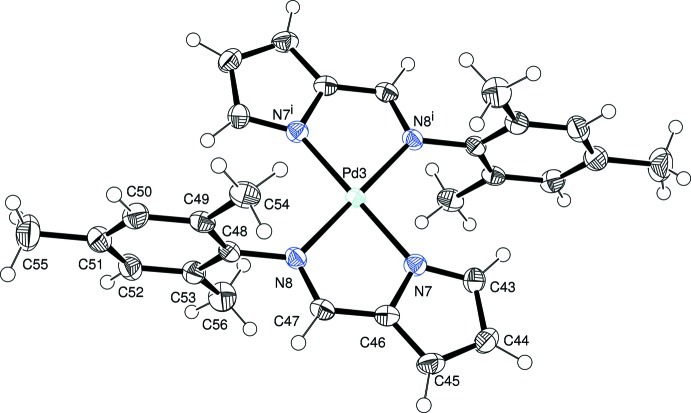
Molecular structure of the molecule with Pd3 being situated at a inversion center with thermal ellipsoids at the 50% probability level (i = 1 - x, -1 - y, -z).

## supplementary crystallographic information

### Comment

In the course of a project related to the supramolecular structures of square planar nickel and palladium complexes of pyrrole-2-carbaldehyde based Schiff base ligands in comparison with the structures of the free ligands the molecular structure of the title compound was determined. The free ligands form centrosymmetric dimers *via* N—H···N hydrogen bonds between the pyrrole NH function and the imine nitrogen atom of a neighboring molecule (Crestani *et al.*, 2011; Gomes *et al.*, 2010; Imhof, 2013).

In the crystal structure three crystallographically independent complexes are observed of which one is located in a general position, whereas the palladium atoms of the other molecules are situated on crystallographic inversion centers (Figures 1–3). The aromatic substituents at the imine nitrogen atoms show dihedral angles of 87.6 (7)° and 83.64 (7)° (molecule 1), 74.3 (7)° (molecule 2) and 88.3 (7)° (molecule 3) with respect to the corresponding PdN_4_ plane. As it is expected bond lengths in the NCCN backbone of the ligands change upon coordination to palladium corresponding to a delocalized formally anionic 1,4-diazadienyl subunit coordinating the metal atoms. Highly related nickel and palladium complexes show similar structural features (Anderson *et al.*, 2006; Bellabarba *et al.*, 2003; Liang *et al.*, 2004; Pérez-Puente *et al.*, 2008).

### Experimental

*N*-((1*H*-Pyrrol-2-yl)methylene)-2,4,6-trimethylaniline (213 mg, 1 mmol) and [Pd(PPh_3_)_4_] (580 mg, 0.5 mmol) were dissolved in 20 ml anhydrous toluene under an argon atmosphere. After the solution is stirred at room temperature for 2 h it was filtered through a short bed of celite. Afterwards the solution was concentrated to *ca* 10 ml *in vacuo*. Crystalline material of the title compound was obtained from this solution after 1 week at -20°C (yield: 214 mg, 81%).

### Refinement

Hydrogen atoms have been included into the refinement in calculated positions (methyl H atoms allowed to rotate but not to tip) with fixed thermal parameter of *U*_iso_(H) = 1.2 *U*_eq_(C) for aromatic C—H groups and the imine C—H function and a thermal parameter of *U*_iso_(H) = 1.5 *U*_eq_(C) for methyl groups.

### Crystal data

**Table:**

[Pd(C_14_H_15_N_2_)_2_]	*Z* = 4
*M**_r_* = 528.96	*F*(000) = 1088
Triclinic, *P*1	*D*_x_ = 1.432 Mg m^−^^3^
Hall symbol: -P 1	Mo *K*α radiation, λ = 0.71073 Å
*a* = 13.4342 (2) Å	Cell parameters from 18633 reflections
*b* = 13.7395 (3) Å	θ = 1.5–27.5°
*c* = 13.9932 (3) Å	µ = 0.78 mm^−^^1^
α = 89.174 (11)°	*T* = 183 K
β = 77.075 (12)°	Cube, yellow
γ = 77.159 (12)°	0.3 × 0.3 × 0.2 mm
*V* = 2452.76 (19) Å^3^	

### Data collection

**Table:**

Nonius KappaCCD diffractometer	9176 reflections with *I* > 2σ(*I*)
Radiation source: fine-focus sealed tube	*R*_int_ = 0.025
Graphite monochromator	θ_max_ = 27.5°, θ_min_ = 1.5°
φ and ω scans	*h* = −17→17
18633 measured reflections	*k* = −17→16
11178 independent reflections	*l* = −17→18

### Refinement

**Table:**

Refinement on *F*^2^	Primary atom site location: structure-invariant direct methods
Least-squares matrix: full	Secondary atom site location: difference Fourier map
*R*[*F*^2^ > 2σ(*F*^2^)] = 0.032	Hydrogen site location: inferred from neighbouring sites
*wR*(*F*^2^) = 0.082	H-atom parameters constrained
*S* = 1.04	*w* = 1/[σ^2^(*F*_o_^2^) + (0.0378*P*)^2^ + 0.9566*P*] where *P* = (*F*_o_^2^ + 2*F*_c_^2^)/3
11178 reflections	(Δ/σ)_max_ = 0.013
610 parameters	Δρ_max_ = 1.17 e Å^−^^3^
0 restraints	Δρ_min_ = −0.80 e Å^−^^3^

### Special details

**Table:**

Geometry. All e.s.d.'s (except the e.s.d. in the dihedral angle between two l.s. planes) are estimated using the full covariance matrix. The cell e.s.d.'s are taken into account individually in the estimation of e.s.d.'s in distances, angles and torsion angles; correlations between e.s.d.'s in cell parameters are only used when they are defined by crystal symmetry. An approximate (isotropic) treatment of cell e.s.d.'s is used for estimating e.s.d.'s involving l.s. planes.
Refinement. Refinement of *F*^2^ against ALL reflections. The weighted *R*-factor *wR* and goodness of fit *S* are based on *F*^2^, conventional *R*-factors *R* are based on *F*, with *F* set to zero for negative *F*^2^. The threshold expression of *F*^2^ > σ(*F*^2^) is used only for calculating *R*-factors(gt) *etc*. and is not relevant to the choice of reflections for refinement. *R*-factors based on *F*^2^ are statistically about twice as large as those based on *F*, and *R*- factors based on ALL data will be even larger.

### Fractional atomic coordinates and isotropic or equivalent isotropic displacement parameters (Å^2^)

**Table:**

	*x*	*y*	*z*	*U*_iso_*/*U*_eq_	
Pd1	0.163237 (12)	0.246906 (13)	0.464295 (12)	0.02049 (6)	
N1	0.21166 (14)	0.30994 (14)	0.33580 (14)	0.0215 (4)	
C1	0.29683 (18)	0.33558 (18)	0.28211 (17)	0.0248 (5)	
H1A	0.3645	0.3190	0.2961	0.030*	
C2	0.27183 (18)	0.39051 (17)	0.20209 (18)	0.0264 (5)	
H2A	0.3185	0.4172	0.1530	0.032*	
C3	0.16522 (18)	0.39838 (17)	0.20851 (18)	0.0252 (5)	
H3A	0.1250	0.4313	0.1648	0.030*	
C4	0.12957 (18)	0.34839 (17)	0.29177 (17)	0.0226 (5)	
N2	0.02318 (14)	0.28818 (14)	0.42391 (14)	0.0221 (4)	
C5	0.03045 (18)	0.33428 (17)	0.34115 (18)	0.0241 (5)	
H5A	−0.0296	0.3577	0.3149	0.029*	
C6	−0.07864 (17)	0.27543 (18)	0.47480 (17)	0.0236 (5)	
C7	−0.14339 (18)	0.35159 (18)	0.53961 (18)	0.0272 (5)	
C8	−0.24287 (19)	0.33935 (19)	0.58602 (19)	0.0314 (6)	
H8A	−0.2885	0.3912	0.6293	0.038*	
C9	−0.27738 (18)	0.2546 (2)	0.57123 (18)	0.0302 (6)	
C10	−0.2091 (2)	0.1790 (2)	0.50884 (19)	0.0325 (6)	
H10A	−0.2312	0.1197	0.4989	0.039*	
C11	−0.10875 (19)	0.18755 (19)	0.46022 (18)	0.0286 (5)	
C12	−0.1079 (2)	0.4439 (2)	0.5595 (2)	0.0452 (7)	
H12A	−0.0394	0.4249	0.5763	0.068*	
H12B	−0.1589	0.4831	0.6142	0.068*	
H12C	−0.1024	0.4840	0.5008	0.068*	
C13	−0.3866 (2)	0.2449 (2)	0.6208 (2)	0.0430 (7)	
H13A	−0.4379	0.3013	0.6041	0.064*	
H13B	−0.3938	0.2449	0.6920	0.064*	
H13C	−0.3988	0.1823	0.5983	0.064*	
C14	−0.0364 (2)	0.1042 (2)	0.3940 (2)	0.0490 (8)	
H14A	0.0308	0.0866	0.4135	0.074*	
H14B	−0.0251	0.1256	0.3261	0.074*	
H14C	−0.0677	0.0458	0.3992	0.074*	
N3	0.11406 (15)	0.18656 (15)	0.59387 (14)	0.0250 (4)	
C15	0.02657 (19)	0.17013 (18)	0.65332 (18)	0.0270 (5)	
H15A	−0.0418	0.1904	0.6413	0.032*	
C16	0.05000 (19)	0.11904 (17)	0.73526 (18)	0.0273 (5)	
H16A	0.0016	0.0992	0.7882	0.033*	
C17	0.1577 (2)	0.10277 (19)	0.72446 (18)	0.0307 (6)	
H17A	0.1975	0.0689	0.7681	0.037*	
C18	0.19615 (18)	0.14559 (19)	0.63729 (18)	0.0281 (5)	
C19	0.29609 (19)	0.1542 (2)	0.58527 (18)	0.0311 (6)	
H19A	0.3566	0.1257	0.6091	0.037*	
N4	0.30427 (15)	0.20230 (16)	0.50320 (15)	0.0267 (4)	
C20	0.40720 (17)	0.20400 (18)	0.44600 (17)	0.0252 (5)	
C21	0.44577 (18)	0.29036 (19)	0.44667 (18)	0.0287 (5)	
C22	0.54514 (19)	0.2896 (2)	0.39070 (18)	0.0302 (5)	
H22A	0.5723	0.3476	0.3915	0.036*	
C23	0.60637 (18)	0.2066 (2)	0.33336 (18)	0.0286 (5)	
C24	0.56480 (19)	0.1234 (2)	0.33281 (19)	0.0310 (6)	
H24A	0.6055	0.0664	0.2935	0.037*	
C25	0.46576 (18)	0.11966 (19)	0.38757 (18)	0.0278 (5)	
C26	0.3817 (2)	0.3823 (2)	0.5075 (2)	0.0424 (7)	
H26A	0.4168	0.4378	0.4918	0.064*	
H26B	0.3747	0.3685	0.5773	0.064*	
H26C	0.3120	0.4002	0.4930	0.064*	
C27	0.7144 (2)	0.2080 (2)	0.2737 (2)	0.0406 (7)	
H27A	0.7564	0.1395	0.2615	0.061*	
H27B	0.7481	0.2467	0.3098	0.061*	
H27C	0.7088	0.2388	0.2109	0.061*	
C28	0.4231 (2)	0.0275 (2)	0.3841 (2)	0.0391 (6)	
H28A	0.4667	−0.0168	0.3290	0.059*	
H28B	0.3510	0.0468	0.3755	0.059*	
H28C	0.4240	−0.0074	0.4456	0.059*	
Pd2	0.0000	−0.5000	0.0000	0.02017 (7)	
N5	−0.09861 (15)	−0.51167 (14)	0.12940 (15)	0.0244 (4)	
C29	−0.16114 (18)	−0.57003 (18)	0.17554 (19)	0.0285 (5)	
H29A	−0.1725	−0.6277	0.1469	0.034*	
C30	−0.20707 (19)	−0.53356 (19)	0.27177 (19)	0.0303 (6)	
H30A	−0.2544	−0.5613	0.3192	0.036*	
C31	−0.17046 (19)	−0.44942 (19)	0.28480 (19)	0.0302 (6)	
H31A	−0.1873	−0.4085	0.3429	0.036*	
C32	−0.10407 (18)	−0.43655 (17)	0.19610 (18)	0.0255 (5)	
C33	−0.04886 (17)	−0.36287 (17)	0.16084 (18)	0.0252 (5)	
H33A	−0.0510	−0.3077	0.2020	0.030*	
N6	0.00543 (14)	−0.37176 (14)	0.07024 (15)	0.0235 (4)	
C34	0.05818 (17)	−0.29237 (16)	0.03709 (17)	0.0219 (5)	
C35	0.01881 (18)	−0.22382 (17)	−0.02786 (18)	0.0247 (5)	
C36	0.06473 (18)	−0.14246 (17)	−0.05187 (18)	0.0255 (5)	
H36A	0.0385	−0.0953	−0.0957	0.031*	
C37	0.14732 (18)	−0.12827 (17)	−0.01385 (17)	0.0249 (5)	
C38	0.18579 (18)	−0.19909 (17)	0.04811 (18)	0.0261 (5)	
H38A	0.2433	−0.1908	0.0737	0.031*	
C39	0.14350 (18)	−0.28187 (17)	0.07435 (18)	0.0254 (5)	
C40	−0.0703 (2)	−0.2361 (2)	−0.0715 (2)	0.0368 (6)	
H40A	−0.1266	−0.2513	−0.0197	0.055*	
H40B	−0.0456	−0.2910	−0.1211	0.055*	
H40C	−0.0969	−0.1742	−0.1022	0.055*	
C41	0.1938 (2)	−0.03800 (19)	−0.0373 (2)	0.0341 (6)	
H41A	0.1422	0.0159	−0.0575	0.051*	
H41B	0.2565	−0.0556	−0.0908	0.051*	
H41C	0.2128	−0.0157	0.0210	0.051*	
C42	0.1889 (2)	−0.3563 (2)	0.1422 (2)	0.0441 (7)	
H42A	0.2078	−0.4234	0.1112	0.066*	
H42B	0.1368	−0.3549	0.2039	0.066*	
H42C	0.2515	−0.3389	0.1555	0.066*	
Pd3	0.5000	0.0000	0.0000	0.02059 (7)	
N7	0.38702 (15)	−0.02108 (14)	0.11542 (14)	0.0245 (4)	
C43	0.35456 (19)	−0.09420 (18)	0.16945 (17)	0.0271 (5)	
H43A	0.3903	−0.1625	0.1626	0.033*	
C44	0.26070 (19)	−0.0556 (2)	0.23723 (19)	0.0318 (6)	
H44A	0.2218	−0.0924	0.2834	0.038*	
C45	0.23511 (19)	0.0468 (2)	0.22423 (18)	0.0316 (6)	
H45A	0.1757	0.0937	0.2599	0.038*	
C46	0.31405 (18)	0.06689 (18)	0.14846 (18)	0.0253 (5)	
C47	0.33221 (18)	0.15209 (17)	0.09806 (18)	0.0257 (5)	
H47A	0.2865	0.2155	0.1170	0.031*	
N8	0.41321 (15)	0.14272 (14)	0.02451 (14)	0.0238 (4)	
C48	0.43127 (17)	0.22895 (16)	−0.02939 (17)	0.0240 (5)	
C49	0.49587 (18)	0.28507 (17)	−0.00173 (18)	0.0255 (5)	
C50	0.5119 (2)	0.36819 (18)	−0.05528 (19)	0.0311 (6)	
H50A	0.5557	0.4071	−0.0376	0.037*	
C51	0.4655 (2)	0.39644 (19)	−0.1345 (2)	0.0345 (6)	
C52	0.4029 (2)	0.33834 (19)	−0.1598 (2)	0.0329 (6)	
H52A	0.3703	0.3572	−0.2129	0.040*	
C53	0.38611 (18)	0.25320 (18)	−0.10975 (18)	0.0271 (5)	
C54	0.5471 (2)	0.2539 (2)	0.0824 (2)	0.0348 (6)	
H54A	0.6007	0.2919	0.0829	0.052*	
H54B	0.5799	0.1824	0.0749	0.052*	
H54C	0.4942	0.2671	0.1443	0.052*	
C55	0.4836 (3)	0.4871 (2)	−0.1916 (2)	0.0506 (8)	
H55A	0.5009	0.4699	−0.2620	0.076*	
H55B	0.5418	0.5097	−0.1743	0.076*	
H55C	0.4200	0.5405	−0.1756	0.076*	
C56	0.3241 (2)	0.1878 (2)	−0.1442 (2)	0.0394 (6)	
H56A	0.2689	0.1764	−0.0892	0.059*	
H56B	0.3707	0.1236	−0.1698	0.059*	
H56C	0.2922	0.2207	−0.1962	0.059*	

### Atomic displacement parameters (Å^2^)

**Table:**

	*U*^11^	*U*^22^	*U*^33^	*U*^12^	*U*^13^	*U*^23^
Pd1	0.01597 (9)	0.02503 (10)	0.02050 (10)	−0.00485 (7)	−0.00405 (7)	0.00239 (7)
N1	0.0179 (9)	0.0202 (9)	0.0258 (10)	−0.0006 (7)	−0.0073 (8)	−0.0013 (8)
C1	0.0224 (11)	0.0279 (12)	0.0239 (12)	−0.0055 (10)	−0.0047 (9)	0.0010 (10)
C2	0.0271 (12)	0.0247 (12)	0.0266 (13)	−0.0071 (10)	−0.0032 (10)	0.0022 (10)
C3	0.0273 (12)	0.0208 (11)	0.0285 (13)	−0.0044 (10)	−0.0094 (10)	0.0020 (9)
C4	0.0236 (11)	0.0206 (11)	0.0241 (12)	−0.0043 (9)	−0.0069 (9)	−0.0005 (9)
N2	0.0170 (9)	0.0246 (10)	0.0256 (11)	−0.0055 (8)	−0.0058 (8)	0.0010 (8)
C5	0.0209 (11)	0.0230 (11)	0.0293 (13)	−0.0039 (9)	−0.0087 (10)	−0.0009 (10)
C6	0.0167 (10)	0.0300 (12)	0.0253 (12)	−0.0059 (9)	−0.0067 (9)	0.0021 (10)
C7	0.0240 (12)	0.0269 (12)	0.0295 (13)	−0.0032 (10)	−0.0058 (10)	−0.0004 (10)
C8	0.0239 (12)	0.0324 (13)	0.0320 (14)	−0.0005 (10)	0.0003 (10)	−0.0002 (11)
C9	0.0202 (11)	0.0425 (15)	0.0279 (13)	−0.0083 (11)	−0.0050 (10)	0.0089 (11)
C10	0.0305 (13)	0.0359 (14)	0.0356 (15)	−0.0171 (11)	−0.0072 (11)	0.0009 (11)
C11	0.0248 (12)	0.0347 (14)	0.0278 (13)	−0.0109 (10)	−0.0048 (10)	−0.0040 (10)
C12	0.0390 (15)	0.0328 (15)	0.059 (2)	−0.0103 (12)	0.0029 (14)	−0.0136 (13)
C13	0.0280 (14)	0.0587 (19)	0.0416 (17)	−0.0144 (13)	−0.0027 (12)	0.0142 (14)
C14	0.0412 (16)	0.0433 (17)	0.059 (2)	−0.0170 (14)	0.0047 (14)	−0.0226 (15)
N3	0.0237 (10)	0.0282 (10)	0.0235 (10)	−0.0063 (8)	−0.0058 (8)	0.0027 (8)
C15	0.0246 (12)	0.0270 (12)	0.0288 (13)	−0.0099 (10)	−0.0009 (10)	−0.0017 (10)
C16	0.0336 (13)	0.0237 (12)	0.0242 (13)	−0.0125 (10)	0.0002 (10)	0.0017 (10)
C17	0.0355 (14)	0.0314 (13)	0.0258 (13)	−0.0087 (11)	−0.0074 (11)	0.0056 (10)
C18	0.0250 (12)	0.0340 (13)	0.0257 (13)	−0.0068 (10)	−0.0068 (10)	0.0062 (10)
C19	0.0242 (12)	0.0412 (15)	0.0293 (14)	−0.0055 (11)	−0.0110 (10)	0.0074 (11)
N4	0.0181 (9)	0.0361 (11)	0.0263 (11)	−0.0058 (8)	−0.0068 (8)	0.0071 (9)
C20	0.0165 (11)	0.0352 (13)	0.0245 (12)	−0.0046 (10)	−0.0076 (9)	0.0075 (10)
C21	0.0229 (12)	0.0362 (14)	0.0263 (13)	−0.0044 (10)	−0.0064 (10)	0.0010 (11)
C22	0.0274 (12)	0.0362 (14)	0.0298 (14)	−0.0134 (11)	−0.0060 (10)	0.0006 (11)
C23	0.0232 (12)	0.0387 (14)	0.0238 (13)	−0.0069 (11)	−0.0053 (10)	0.0025 (10)
C24	0.0259 (12)	0.0347 (14)	0.0293 (14)	−0.0026 (11)	−0.0037 (10)	−0.0023 (11)
C25	0.0235 (12)	0.0331 (13)	0.0290 (13)	−0.0063 (10)	−0.0107 (10)	0.0052 (10)
C26	0.0331 (14)	0.0431 (16)	0.0480 (18)	−0.0103 (13)	−0.0008 (13)	−0.0081 (13)
C27	0.0271 (13)	0.0516 (17)	0.0391 (16)	−0.0104 (12)	0.0023 (12)	−0.0009 (13)
C28	0.0321 (14)	0.0371 (15)	0.0499 (18)	−0.0107 (12)	−0.0097 (13)	0.0010 (13)
Pd2	0.01905 (12)	0.01752 (12)	0.02423 (14)	−0.00706 (9)	−0.00241 (10)	−0.00192 (9)
N5	0.0224 (10)	0.0228 (10)	0.0270 (11)	−0.0076 (8)	−0.0012 (8)	−0.0013 (8)
C29	0.0246 (12)	0.0249 (12)	0.0362 (14)	−0.0088 (10)	−0.0042 (10)	0.0009 (10)
C30	0.0238 (12)	0.0318 (13)	0.0327 (14)	−0.0077 (10)	0.0003 (10)	0.0059 (11)
C31	0.0275 (12)	0.0304 (13)	0.0288 (14)	−0.0039 (10)	−0.0003 (10)	−0.0039 (10)
C32	0.0230 (11)	0.0237 (12)	0.0281 (13)	−0.0050 (9)	−0.0024 (10)	−0.0049 (10)
C33	0.0235 (11)	0.0231 (12)	0.0283 (13)	−0.0052 (9)	−0.0042 (10)	−0.0063 (10)
N6	0.0228 (10)	0.0204 (9)	0.0288 (11)	−0.0076 (8)	−0.0055 (8)	−0.0026 (8)
C34	0.0229 (11)	0.0160 (10)	0.0262 (12)	−0.0058 (9)	−0.0025 (9)	−0.0044 (9)
C35	0.0252 (12)	0.0219 (11)	0.0268 (13)	−0.0040 (9)	−0.0064 (10)	−0.0049 (9)
C36	0.0298 (12)	0.0199 (11)	0.0265 (13)	−0.0041 (10)	−0.0076 (10)	0.0027 (9)
C37	0.0288 (12)	0.0199 (11)	0.0256 (13)	−0.0078 (10)	−0.0030 (10)	−0.0004 (9)
C38	0.0245 (12)	0.0273 (12)	0.0296 (13)	−0.0111 (10)	−0.0074 (10)	0.0003 (10)
C39	0.0233 (11)	0.0247 (12)	0.0298 (13)	−0.0077 (10)	−0.0070 (10)	0.0032 (10)
C40	0.0367 (14)	0.0328 (14)	0.0473 (17)	−0.0091 (12)	−0.0217 (13)	0.0018 (12)
C41	0.0396 (14)	0.0268 (13)	0.0386 (15)	−0.0151 (11)	−0.0070 (12)	0.0052 (11)
C42	0.0391 (15)	0.0451 (17)	0.059 (2)	−0.0195 (13)	−0.0251 (14)	0.0239 (15)
Pd3	0.01993 (12)	0.01823 (12)	0.02178 (13)	−0.00241 (9)	−0.00277 (10)	0.00014 (9)
N7	0.0244 (10)	0.0237 (10)	0.0240 (11)	−0.0041 (8)	−0.0035 (8)	−0.0016 (8)
C43	0.0310 (13)	0.0271 (12)	0.0243 (13)	−0.0075 (10)	−0.0071 (10)	0.0023 (10)
C44	0.0292 (13)	0.0385 (14)	0.0286 (14)	−0.0133 (11)	−0.0031 (11)	0.0057 (11)
C45	0.0236 (12)	0.0393 (14)	0.0270 (13)	−0.0026 (11)	0.0002 (10)	−0.0027 (11)
C46	0.0213 (11)	0.0272 (12)	0.0259 (13)	−0.0030 (10)	−0.0042 (9)	−0.0029 (10)
C47	0.0223 (11)	0.0230 (12)	0.0299 (13)	−0.0008 (9)	−0.0063 (10)	−0.0029 (10)
N8	0.0237 (10)	0.0188 (9)	0.0278 (11)	−0.0021 (8)	−0.0064 (8)	0.0003 (8)
C48	0.0226 (11)	0.0184 (11)	0.0277 (13)	−0.0005 (9)	−0.0027 (10)	−0.0009 (9)
C49	0.0219 (11)	0.0233 (12)	0.0292 (13)	−0.0010 (9)	−0.0054 (10)	−0.0048 (10)
C50	0.0303 (13)	0.0250 (12)	0.0377 (15)	−0.0094 (10)	−0.0037 (11)	−0.0041 (11)
C51	0.0340 (14)	0.0254 (13)	0.0398 (16)	−0.0036 (11)	−0.0027 (12)	0.0013 (11)
C52	0.0357 (14)	0.0300 (13)	0.0326 (14)	−0.0039 (11)	−0.0106 (11)	0.0060 (11)
C53	0.0264 (12)	0.0247 (12)	0.0310 (13)	−0.0058 (10)	−0.0078 (10)	−0.0009 (10)
C54	0.0304 (13)	0.0380 (15)	0.0366 (15)	−0.0048 (11)	−0.0115 (11)	−0.0021 (12)
C55	0.062 (2)	0.0334 (16)	0.057 (2)	−0.0176 (15)	−0.0088 (16)	0.0127 (14)
C56	0.0426 (16)	0.0416 (16)	0.0424 (17)	−0.0166 (13)	−0.0199 (13)	0.0019 (13)

### Geometric parameters (Å, º)

**Table:**

Pd1—N3	2.0189 (19)	Pd2—N6^i^	2.0530 (19)
Pd1—N1	2.0220 (19)	Pd2—N6	2.0531 (19)
Pd1—N2	2.0429 (18)	N5—C29	1.344 (3)
Pd1—N4	2.0488 (19)	N5—C32	1.380 (3)
N1—C1	1.336 (3)	C29—C30	1.398 (4)
N1—C4	1.382 (3)	C29—H29A	0.9500
C1—C2	1.403 (3)	C30—C31	1.382 (4)
C1—H1A	0.9500	C30—H30A	0.9500
C2—C3	1.395 (3)	C31—C32	1.391 (3)
C2—H2A	0.9500	C31—H31A	0.9500
C3—C4	1.390 (3)	C32—C33	1.405 (3)
C3—H3A	0.9500	C33—N6	1.306 (3)
C4—C5	1.410 (3)	C33—H33A	0.9500
N2—C5	1.307 (3)	N6—C34	1.445 (3)
N2—C6	1.441 (3)	C34—C39	1.396 (3)
C5—H5A	0.9500	C34—C35	1.401 (3)
C6—C11	1.387 (3)	C35—C36	1.395 (3)
C6—C7	1.394 (3)	C35—C40	1.501 (3)
C7—C8	1.395 (3)	C36—C37	1.384 (3)
C7—C12	1.500 (4)	C36—H36A	0.9500
C8—C9	1.380 (4)	C37—C38	1.385 (3)
C8—H8A	0.9500	C37—C41	1.509 (3)
C9—C10	1.388 (4)	C38—C39	1.390 (3)
C9—C13	1.510 (3)	C38—H38A	0.9500
C10—C11	1.397 (3)	C39—C42	1.507 (3)
C10—H10A	0.9500	C40—H40A	0.9800
C11—C14	1.499 (4)	C40—H40B	0.9800
C12—H12A	0.9800	C40—H40C	0.9800
C12—H12B	0.9800	C41—H41A	0.9800
C12—H12C	0.9800	C41—H41B	0.9800
C13—H13A	0.9800	C41—H41C	0.9800
C13—H13B	0.9800	C42—H42A	0.9800
C13—H13C	0.9800	C42—H42B	0.9800
C14—H14A	0.9800	C42—H42C	0.9800
C14—H14B	0.9800	Pd3—N7	2.0202 (19)
C14—H14C	0.9800	Pd3—N7^ii^	2.0203 (19)
N3—C15	1.341 (3)	Pd3—N8	2.0363 (18)
N3—C18	1.384 (3)	Pd3—N8^ii^	2.0363 (18)
C15—C16	1.395 (3)	N7—C43	1.340 (3)
C15—H15A	0.9500	N7—C46	1.386 (3)
C16—C17	1.388 (3)	C43—C44	1.397 (3)
C16—H16A	0.9500	C43—H43A	0.9500
C17—C18	1.390 (3)	C44—C45	1.392 (4)
C17—H17A	0.9500	C44—H44A	0.9500
C18—C19	1.406 (3)	C45—C46	1.394 (3)
C19—N4	1.312 (3)	C45—H45A	0.9500
C19—H19A	0.9500	C46—C47	1.398 (3)
N4—C20	1.439 (3)	C47—N8	1.304 (3)
C20—C21	1.397 (4)	C47—H47A	0.9500
C20—C25	1.402 (4)	N8—C48	1.432 (3)
C21—C22	1.386 (3)	C48—C53	1.396 (3)
C21—C26	1.510 (4)	C48—C49	1.398 (3)
C22—C23	1.391 (4)	C49—C50	1.387 (3)
C22—H22A	0.9500	C49—C54	1.503 (3)
C23—C24	1.380 (4)	C50—C51	1.399 (4)
C23—C27	1.508 (3)	C50—H50A	0.9500
C24—C25	1.390 (3)	C51—C52	1.382 (4)
C24—H24A	0.9500	C51—C55	1.506 (4)
C25—C28	1.507 (4)	C52—C53	1.390 (3)
C26—H26A	0.9800	C52—H52A	0.9500
C26—H26B	0.9800	C53—C56	1.504 (4)
C26—H26C	0.9800	C54—H54A	0.9800
C27—H27A	0.9800	C54—H54B	0.9800
C27—H27B	0.9800	C54—H54C	0.9800
C27—H27C	0.9800	C55—H55A	0.9800
C28—H28A	0.9800	C55—H55B	0.9800
C28—H28B	0.9800	C55—H55C	0.9800
C28—H28C	0.9800	C56—H56A	0.9800
Pd2—N5^i^	2.0180 (19)	C56—H56B	0.9800
Pd2—N5	2.0180 (19)	C56—H56C	0.9800
			
N3—Pd1—N1	178.92 (8)	N5^i^—Pd2—N6	99.19 (8)
N3—Pd1—N2	99.02 (8)	N5—Pd2—N6	80.82 (8)
N1—Pd1—N2	80.86 (7)	N6^i^—Pd2—N6	180.0
N3—Pd1—N4	80.96 (8)	C29—N5—C32	106.6 (2)
N1—Pd1—N4	99.19 (7)	C29—N5—Pd2	141.48 (18)
N2—Pd1—N4	178.66 (8)	C32—N5—Pd2	111.90 (15)
C1—N1—C4	107.20 (19)	N5—C29—C30	110.2 (2)
C1—N1—Pd1	140.47 (16)	N5—C29—H29A	124.9
C4—N1—Pd1	111.95 (14)	C30—C29—H29A	124.9
N1—C1—C2	110.1 (2)	C31—C30—C29	107.0 (2)
N1—C1—H1A	124.9	C31—C30—H30A	126.5
C2—C1—H1A	124.9	C29—C30—H30A	126.5
C3—C2—C1	106.8 (2)	C30—C31—C32	106.5 (2)
C3—C2—H2A	126.6	C30—C31—H31A	126.8
C1—C2—H2A	126.6	C32—C31—H31A	126.8
C4—C3—C2	106.2 (2)	N5—C32—C31	109.8 (2)
C4—C3—H3A	126.9	N5—C32—C33	116.1 (2)
C2—C3—H3A	126.9	C31—C32—C33	134.1 (2)
N1—C4—C3	109.68 (19)	N6—C33—C32	118.6 (2)
N1—C4—C5	115.9 (2)	N6—C33—H33A	120.7
C3—C4—C5	134.3 (2)	C32—C33—H33A	120.7
C5—N2—C6	118.08 (19)	C33—N6—C34	116.21 (19)
C5—N2—Pd1	112.88 (15)	C33—N6—Pd2	112.33 (15)
C6—N2—Pd1	129.02 (15)	C34—N6—Pd2	131.46 (15)
N2—C5—C4	118.4 (2)	C39—C34—C35	121.1 (2)
N2—C5—H5A	120.8	C39—C34—N6	119.7 (2)
C4—C5—H5A	120.8	C35—C34—N6	119.1 (2)
C11—C6—C7	121.8 (2)	C36—C35—C34	118.2 (2)
C11—C6—N2	119.6 (2)	C36—C35—C40	120.0 (2)
C7—C6—N2	118.6 (2)	C34—C35—C40	121.8 (2)
C8—C7—C6	117.8 (2)	C37—C36—C35	122.0 (2)
C8—C7—C12	120.7 (2)	C37—C36—H36A	119.0
C6—C7—C12	121.5 (2)	C35—C36—H36A	119.0
C9—C8—C7	122.3 (2)	C36—C37—C38	118.0 (2)
C9—C8—H8A	118.8	C36—C37—C41	121.6 (2)
C7—C8—H8A	118.8	C38—C37—C41	120.4 (2)
C8—C9—C10	118.0 (2)	C37—C38—C39	122.6 (2)
C8—C9—C13	120.9 (2)	C37—C38—H38A	118.7
C10—C9—C13	121.1 (3)	C39—C38—H38A	118.7
C9—C10—C11	122.0 (2)	C38—C39—C34	118.0 (2)
C9—C10—H10A	119.0	C38—C39—C42	120.0 (2)
C11—C10—H10A	119.0	C34—C39—C42	122.0 (2)
C6—C11—C10	118.0 (2)	C35—C40—H40A	109.5
C6—C11—C14	121.3 (2)	C35—C40—H40B	109.5
C10—C11—C14	120.7 (2)	H40A—C40—H40B	109.5
C7—C12—H12A	109.5	C35—C40—H40C	109.5
C7—C12—H12B	109.5	H40A—C40—H40C	109.5
H12A—C12—H12B	109.5	H40B—C40—H40C	109.5
C7—C12—H12C	109.5	C37—C41—H41A	109.5
H12A—C12—H12C	109.5	C37—C41—H41B	109.5
H12B—C12—H12C	109.5	H41A—C41—H41B	109.5
C9—C13—H13A	109.5	C37—C41—H41C	109.5
C9—C13—H13B	109.5	H41A—C41—H41C	109.5
H13A—C13—H13B	109.5	H41B—C41—H41C	109.5
C9—C13—H13C	109.5	C39—C42—H42A	109.5
H13A—C13—H13C	109.5	C39—C42—H42B	109.5
H13B—C13—H13C	109.5	H42A—C42—H42B	109.5
C11—C14—H14A	109.5	C39—C42—H42C	109.5
C11—C14—H14B	109.5	H42A—C42—H42C	109.5
H14A—C14—H14B	109.5	H42B—C42—H42C	109.5
C11—C14—H14C	109.5	N7—Pd3—N7^ii^	180.0
H14A—C14—H14C	109.5	N7—Pd3—N8	80.54 (8)
H14B—C14—H14C	109.5	N7^ii^—Pd3—N8	99.46 (8)
C15—N3—C18	106.8 (2)	N7—Pd3—N8^ii^	99.46 (8)
C15—N3—Pd1	141.05 (17)	N7^ii^—Pd3—N8^ii^	80.54 (8)
C18—N3—Pd1	112.17 (15)	N8—Pd3—N8^ii^	180.0
N3—C15—C16	110.5 (2)	C43—N7—C46	106.89 (19)
N3—C15—H15A	124.8	C43—N7—Pd3	140.86 (16)
C16—C15—H15A	124.8	C46—N7—Pd3	112.13 (15)
C17—C16—C15	106.7 (2)	N7—C43—C44	110.3 (2)
C17—C16—H16A	126.7	N7—C43—H43A	124.8
C15—C16—H16A	126.7	C44—C43—H43A	124.8
C16—C17—C18	106.7 (2)	C45—C44—C43	107.0 (2)
C16—C17—H17A	126.7	C45—C44—H44A	126.5
C18—C17—H17A	126.7	C43—C44—H44A	126.5
N3—C18—C17	109.4 (2)	C44—C45—C46	106.3 (2)
N3—C18—C19	115.7 (2)	C44—C45—H45A	126.9
C17—C18—C19	134.9 (2)	C46—C45—H45A	126.9
N4—C19—C18	118.8 (2)	N7—C46—C45	109.5 (2)
N4—C19—H19A	120.6	N7—C46—C47	115.5 (2)
C18—C19—H19A	120.6	C45—C46—C47	134.9 (2)
C19—N4—C20	118.7 (2)	N8—C47—C46	118.6 (2)
C19—N4—Pd1	112.22 (16)	N8—C47—H47A	120.7
C20—N4—Pd1	128.30 (15)	C46—C47—H47A	120.7
C21—C20—C25	121.0 (2)	C47—N8—C48	119.22 (19)
C21—C20—N4	119.8 (2)	C47—N8—Pd3	113.16 (15)
C25—C20—N4	119.1 (2)	C48—N8—Pd3	127.61 (15)
C22—C21—C20	118.4 (2)	C53—C48—C49	121.7 (2)
C22—C21—C26	120.5 (2)	C53—C48—N8	119.1 (2)
C20—C21—C26	121.1 (2)	C49—C48—N8	119.1 (2)
C21—C22—C23	122.2 (2)	C50—C49—C48	117.9 (2)
C21—C22—H22A	118.9	C50—C49—C54	121.9 (2)
C23—C22—H22A	118.9	C48—C49—C54	120.2 (2)
C24—C23—C22	117.8 (2)	C49—C50—C51	122.0 (2)
C24—C23—C27	121.3 (2)	C49—C50—H50A	119.0
C22—C23—C27	120.9 (2)	C51—C50—H50A	119.0
C23—C24—C25	122.6 (2)	C52—C51—C50	118.2 (2)
C23—C24—H24A	118.7	C52—C51—C55	120.6 (3)
C25—C24—H24A	118.7	C50—C51—C55	121.2 (3)
C24—C25—C20	118.0 (2)	C51—C52—C53	122.0 (2)
C24—C25—C28	120.8 (2)	C51—C52—H52A	119.0
C20—C25—C28	121.2 (2)	C53—C52—H52A	119.0
C21—C26—H26A	109.5	C52—C53—C48	118.2 (2)
C21—C26—H26B	109.5	C52—C53—C56	120.3 (2)
H26A—C26—H26B	109.5	C48—C53—C56	121.5 (2)
C21—C26—H26C	109.5	C49—C54—H54A	109.5
H26A—C26—H26C	109.5	C49—C54—H54B	109.5
H26B—C26—H26C	109.5	H54A—C54—H54B	109.5
C23—C27—H27A	109.5	C49—C54—H54C	109.5
C23—C27—H27B	109.5	H54A—C54—H54C	109.5
H27A—C27—H27B	109.5	H54B—C54—H54C	109.5
C23—C27—H27C	109.5	C51—C55—H55A	109.5
H27A—C27—H27C	109.5	C51—C55—H55B	109.5
H27B—C27—H27C	109.5	H55A—C55—H55B	109.5
C25—C28—H28A	109.5	C51—C55—H55C	109.5
C25—C28—H28B	109.5	H55A—C55—H55C	109.5
H28A—C28—H28B	109.5	H55B—C55—H55C	109.5
C25—C28—H28C	109.5	C53—C56—H56A	109.5
H28A—C28—H28C	109.5	C53—C56—H56B	109.5
H28B—C28—H28C	109.5	H56A—C56—H56B	109.5
N5^i^—Pd2—N5	179.999 (2)	C53—C56—H56C	109.5
N5^i^—Pd2—N6^i^	80.82 (8)	H56A—C56—H56C	109.5
N5—Pd2—N6^i^	99.18 (8)	H56B—C56—H56C	109.5

Symmetry codes: (i) −*x*, −*y*−1, −*z*; (ii) −*x*+1, −*y*, −*z*.
